# The Neuroscience of Decision-Making: Linking the Brain, Behavior, and Society

**DOI:** 10.3390/brainsci16060574

**Published:** 2026-05-28

**Authors:** Bruno Kluwe-Schiavon, Giorgio Luigi Manenti

**Affiliations:** 1 Department of Psychiatry and Behavioral Sciences, McGovern Medical School, University of Texas Health Science Center at Houston, Houston, TX 77054, USA; 2 Postgraduate Program in Health Sciences, Universidade do Extremo Sul Catarinense, Criciúma 88806-000, Brazil; 3 Institute of Systems Neuroscience, University Medical Center Hamburg-Eppendorf, 20246 Hamburg, Germany; g.manenti@uke.de

The capacity to choose among competing alternatives is one of the most fundamental and pervasive features of human cognition, yet also among the most conceptually complex and methodologically unresolved problems in cognitive neuroscience. Decision-making is increasingly recognized as a dynamic and multidimensional process rather than a unitary cognitive function, recruiting overlapping yet dissociable neural systems operating across timescales ranging from milliseconds, such as automatic motor tendencies, to years, such as the gradual consolidation of environmental habits [1,2,3]. Thus, choices emerge through the continuous interaction between neural, cognitive, emotional, social, and environmental factors, resulting in behavioral outcomes that vary substantially across contexts, timescales, and individual experiences. The same individual may exhibit different decisions depending on situational demands, social pressure, motivational states, prior learning, or subtle contextual manipulations such as persuasive communication, advertising exposure, or interpersonal influence [4,5,6,7,8]. Importantly, subjective preferences themselves are not fixed entities, but flexible constructs continuously shaped and renegotiated through interactions with external environments, social dynamics, and contextual constraints [6,7]. Human choices therefore often emerge from the dynamic arbitration between automatic motivational impulses and deliberative regulatory mechanisms [9,10], whose relative influence depends strongly on contextual and interpersonal conditions [11,12].

From this perspective, decision-making reflects the plasticity of human behavior, revealing how internal dispositions, such as personality traits, attentional states, moral values, or prosocial orientations, can be reinforced, modulated, or even overridden by contextual and environmental pressures. These dynamics become particularly evident in domains involving consumer behavior, social alignment, moral persuasion, and sustainable decision-making, where choices emerge through the continuous negotiation between internal preferences, immediate rewards, social influences, and collective long-term outcomes. Rather than reflecting stable internal coherence alone, adaptive behavior may therefore arise from strategic adjustments to ecological, economic, and interpersonal demands. Understanding decision-making consequently requires an integrative framework capable of linking neural mechanisms with behavior and the broader societal environments in which choices occur. Integrating individual-level neuroscience with the social and environmental forces shaping choice is therefore not merely a theoretical supplement, but a methodological requirement. In line with this perspective, this Special Issue, entitled “The Neuroscience of Decision-Making: Linking the Brain, Behavior, and Society,” brings together five original contributions investigating decision-making across diverse domains, including everyday choices, moral deliberation, social interaction, sustainability, and adaptive behavior. Specifically, through complementary approaches spanning behavioral experimentation, neurophysiology, neuroimaging, computational modeling, and clinical research, the collected studies explore how neural systems dynamically interact with contextual and social factors to shape human preferences, actions, and behavioral adaptation.

Across the contributions collected in this Special Issue, there is a recurring theme concerning how internal dispositions, regulatory processes, and subjective preferences are continuously shaped, constrained, and sometimes overridden by contextual and interpersonal forces. Baquedano and colleagues examined this directly in the context of consumer behavior, asking whether a brief mindfulness instruction could attenuate the psychophysiological responses elicited by food advertising (contribution 1). In a randomized experiment with sixty participants, one group received a dereification instruction, framing stimuli as transient mental events, before completing an approach–avoidance task while EEG recordings, salivation measures, and self-reports were collected alongside event-related potentials. The authors found that the mindfulness instruction had no measurable effect on approach tendencies, craving, hunger, or salivation, and the N1, N2, P3, and late positive potential components were equally unaltered across groups. Nevertheless, what was consistent across conditions was the amplifying effect of advertising itself: commercial food images increased the appetitive appeal of neutral foods, indexed by elevated N2, P3, and late positive potential responses, regardless of the preceding instruction. These results align with the broader literature showing that brief regulatory instructions are frequently insufficient to override automatic consummatory responses to salient motivational stimuli, particularly when the regulatory load is low [13]. Their study also suggests that if shallow attentional modulation cannot buffer the neural impact of food advertising, population-level strategies may need to target the advertising environment directly rather than relying only on individual-level coping instructions.

In another study, Uribe, Ávila-Chauvet, and Mejía investigated how stable individual traits interact with situational demands to shape behavioral choices by using a human analog of the producer–scrounger game (contribution 2). Forty-five participants completed the Guaymas Foraging Task under two cost conditions, zero and eight seconds of search cost for the producer role, while also completing the Big Five Inventory and the Antisocial Process Screening Device. Personality dimensions, including openness, agreeableness, extraversion, and the higher-order metatraits of stability and plasticity, each predicted higher producer indexes, but only under the low-cost condition; as production became more costly, personality-based differences in foraging strategy largely disappeared. Participants above the Antisocial Process Screening Device cutoff scrounged more, again restricted to the low-cost environment. This pattern extends to human social foraging, supporting the well-established finding from behavioral ecology that individual differences operate within the latitude that environmental contingencies permit; when the costs of a preferred strategy become prohibitive, phenotypic variability in behavior contracts [14]. Hence, future studies exposing participants to a single cost level, or treating environmental contingencies as background rather than as a variable, may miss the precise conditions under which trait–behavior associations exist at all.

Where Uribe, Ávila-Chauvet, and Mejía examine how traits shape responses to social environments, Sobeh and Shamay-Tsoory investigated how individuals decide whether to align with others at all (contribution 3). Their theoretical review proposes a goal-directed model of social alignment formalized as a prediction-error minimization process over the gap between self and other, augmented by a meta-learning layer in which the learning rate is adaptively tuned according to the inferred value of aligning versus maintaining independence. The review emphasizes the central regulatory role of the mentalizing network, specifically the dorsomedial prefrontal cortex and temporoparietal junction, which exerts top-down control via the executive network, including the dorsolateral prefrontal cortex and right inferior frontal gyrus, over observation–execution circuitry. This framework shifts the focus from alignment as reflexive imitation to alignment as decision-making under uncertainty. The central claim is that people do not simply synchronize with others because the opportunity is present. Rather, they evaluate whether alignment is useful, with whom they should align, and whether the context makes alignment adaptive. Prior theories of imitation and synchrony explain many of the mechanisms through which alignment emerges, but they give less attention to the arbitration process that determines whether alignment should occur. The proposed model also interprets groupthink and false information cascades as failures of meta-learning and generates testable predictions for developmental, clinical, and neurophysiological research.

Extending this perspective from individual adaptation to interpersonal negotiation, Allegretta, Daffinà, and Balconi examined how moral decisions emerge through live persuasive interaction between individuals (contribution 4). Using EEG hyperscanning to simultaneously capture neural activity across interacting dyads, the study investigated how social influence, emotional engagement, and interpersonal asymmetries shape the revision or maintenance of moral viewpoints. Fourteen dyads, with members assigned the roles of persuasive agent and persuasion target, discussed a moral dilemma while neural activity was recorded simultaneously from both participants. The dyads were categorized by degree of viewpoint change as high, mixed, or low, and inter-individual EEG dissimilarity was analyzed across frequency bands and scalp regions. The authors found a significant increase in frontal delta-band dissimilarity in mixed dyads, compared to both temporo-central and parieto-occipital regions. Because delta-band activity has been associated with motivational and emotional regulation [15,16], frontal differences in this frequency band may help clarify why persuasion affected only one member of the dyad. The authors suggest that this frontal divergence in the delta band may reflect an asymmetrical engagement with the interaction, especially in partially persuaded dyads. In this sense, participants who altered their stance may have become more emotionally attuned to their persuasive partner, while those who maintained their original view may have processed the same moral arguments from a distinct motivational perspective. Allegretta, Daffinà, and Balconi’s study shifts attention from persuasion as an individual decision to persuasion as an interpersonal process in which moral views are challenged, defended, and sometimes revised.

While the preceding studies focused on how contextual and social pressures shape individual and interpersonal decisions, Richelli, Arioli, and Canessa expanded this perspective to choices involving collective welfare and future-oriented outcomes (contribution 5). In scoping review synthesizing evidence from thirty studies, the authors examined the neurocognitive bases of sustainable decision-making and pro-environmental behavior. The authors identify three main neural patterns in sustainable decision-making: perspective-taking and moral cognition, integration of personal and contextual information, and attentional and emotional–motivational processing. Together, these patterns indicate that sustainable choices depend not only on moral concern for others and future generations, but also on the ability to weigh contextual trade-offs and assign motivational value to long-term environmental outcomes. The review also shows that sustainable decision-making may be shaped by some of the same factors involved in prosocial and cooperative behavior, meaning that neural and cognitive processes supporting other choices may also be relevant to sustainability contexts. Framing pro-environmental behavior as cooperative decision-making links the sustainable decision-making literature to neuroeconomics and social neuroscience, two fields that have long examined how people balance self-interest against collective outcomes.

The five contributions in this Special Issue do not converge on a single mechanism of decision-making. Rather, their collective value lies in demonstrating why no isolated mechanism is likely to fully explain how choices emerge across contexts. Across these studies, decisions are shaped by the continuous interaction between internal dispositions and external constraints: personality traits predict behavior only under specific environmental costs (contribution 2), prediction errors influence alignment when social adaptation is advantageous (contribution 3), and persuasion depends not only on moral reasoning but also on the emotional and interpersonal dynamics unfolding between individuals (contribution 4). A similar pattern emerges in the domains of consumer behavior and sustainability. Regulatory strategies may prove insufficient when appetitive or motivational cues are highly salient (contribution 1), while pro-environmental choices depend on the integration of individual preferences with broader collective and future-oriented concerns (contribution 5).

Taken together, these contributions support a view of decision-making not as a fixed or isolated cognitive function, but as a dynamic and context-sensitive process emerging from the ongoing negotiation between brain, behavior, and society. 
